# Supplementary material for: What are the top priorities of patients and clinicians for the organization of primary cardiovascular care in Quebec? A modified e-Delphi study
Source: PLoS One. 2023 Jan 4;18(1):e0280051. doi: 10.1371/journal.pone.0280051 (PMC9812320; doi:10.1371/journal.pone.0280051)
Supplement: S1 Table — (DOCX) [file pone.0280051.s001.docx]

**S1 Table.** **Quantitative results for the organizational items assessed during round two (n=33 panelists).**

| **Organizational items** | **Initial consensus**^a^ | **Difference**^b^ **between [P]atients and [C]linicians** | **Kept in last round?** |
| --- | --- | --- | --- |
| ACCESSIBILITY | | | |
| {A1} Being able to get an appointment with your family doctor on short notice | 81.3% | N.S.  (p = 1.000) | **YES** |
| {A2} Being able to reach a healthcare professional within 24-48 hours in the event of a problem, either on site, by phone, videoconference or email | 87.5% | N.S.  (p = 1.000) | **YES** |
| {A3} Having the option to get longer consultations | 31.3% | N.S.  (p = 0.265) | NO |
| {A4} Having access to all clinic services in the evening and on weekends | 53.1% | N.S.  (p = 0.287) | NO |
| {A5} Having access to all clinic services in French or English | 43.8% | N.S.  (p = 0.735) | NO |
| {A6} Being seen on time for an appointment with little or no delay | 50.0% | N.S.  (p = 0.479) | NO |
| {A7} Free parking near the clinic | 34.4% | N.S.  (p = 1.000) | NO |
| SERVICES NETWORK | | | |
| {SN1} Obtaining short delays for examinations and consultations that must be done outside the clinic | 68.8% | N.S.  (p = 0.128) | NO |
| {SN2} Having access to inexpensive or free resources and programs to improve the health and lifestyle of people with a cardiovascular health condition | 53.1% | N.S.  (p = 0.178) | NO |
| {SN3} Having access to a variety of tests (blood tests, echocardiography, etc.) at the clinic without having to be referred externally | 62.5% | N.S.  (p = 0.726) | NO |
| {SN4} Coordinating the appointments (in and out of the clinic) to minimize the inconvenience to patients | 78.1% | N.S.  (p = 0.209) | **YES** |
| {SN5} Explaining the role of each healthcare professional and when/how to refer to the right person | 50.0% | N.S.  (p = 1.000) | NO |
| CARE AND FOLLOW-UP | | | |
| {CFU1} Having protocols in place to systematically direct patients to the right care and services based on their condition | 53.1% | N.S.  (p = 0.074) | NO |
| {CFU2} Conducting regular follow-ups on the progress made or not (e.g., in a logbook detailing steps of care) | 37.5% | N.S.  (p = 0.291) | NO |
| {CFU3} Offering activities at the clinic on healthy lifestyle and prevention of cardiovascular health problems | 37.5% | **P [17.6%]**  **<**  **C [60.0%]**  **(p = 0.027)** | NO |
| {CFU4} Offering help in managing health-related stress and anxiety | 50.0% | N.S.  (p = 0.479) | NO |
| SELF-MANAGEMENT SUPPORT | | | |
| {SMS1} Receiving general information on cardiovascular health and available support resources | 15.6% | N.S.  (p = 0.645) | NO |
| {SMS2} Receiving personalized information on your own cardiovascular health (personal check-up, origin and nature of the problem, risks, etc.) | 81.3% | N.S.  (p = 1.000) | **YES** |
| {SMS3} Receiving training and tools to help you manage your own health (how to take your blood pressure, what to do based on your results, etc.) | 59.4% | N.S.  (p = 1.000) | NO |
| {SMS4} Receiving practical help to initiate lifestyle changes (nutritional evaluation, health literacy education service, etc.) | 62.5% | N.S.  (p = 0.076) | NO |
| CLINICAL TEAM COMPOSITION | | | |
| {CTC1} Having a pharmacist available on the clinical team | 40.6% | N.S.  (p = 0.070) | NO |
| {CTC2} Having a nutrition specialist available on the clinical team | 68.8% | N.S.  (p = 0.712) | NO |
| {CTC3} Having a physical activity specialist available on the clinical team | 34.4% | N.S.  (p = 0.472) | NO |
| {CTC4} Having a specialist in weight and obesity management available on the clinical team | 53.1% | N.S.  (p = 0.502 | NO |
| {CTC5} Having a smoking cessation specialist available on the clinical team | 31.3% | N.S.  (p = 1.000) | NO |
| {CTC6} Having a nurse specialized in cardiovascular health available on the clinical team | 65.6% | N.S.  (p = 0.712) | NO |
| PROFESSIONAL COLLABORATION | | | |
| {PC1} Ensuring effective collaboration between family doctors and nurses at the clinic | 84.8% | N.S.  (p = 0.335) | **YES** |
| {PC2} Ensuring effective collaboration between the clinic and pharmacists in the community | 63.6% | N.S.  (p = 0.721) | NO |
| {PC3} Ensuring effective collaboration between family doctors and allied healthcare professionals specializing in healthy lifestyles | 66.7% | N.S.  (p = 0.721) | NO |
| {PC4} Ensuring effective collaboration between the clinic and specialist physicians (e.g., cardiologists) | 72.7% | N.S.  (p = 0.057) | **YES** |
| {PC5} Ensuring effective collaboration between the clinic and community resources | 36.4% | N.S.  (p = 0.721) | NO |
| PROFESSIONAL TRAINING | | | |
| {PT1} Healthcare professionals having up-to-date cardiovascular health training in their respective fields | 87.5% | N.S.  (p = 0.319) | **YES** |
| {PT2} Having a doctor who is better trained to provide counseling on nutrition and physical activity | 43.8% | N.S.  (p = 1.000) | NO |
| {PT3} Having a doctor with sufficient clinical experience in cardiovascular health | 56.3% | N.S.  (p = 0.735) | NO |
| PATIENT-PROFESSIONAL RELATIONSHIP | | | |
| {PPR1} Feeling that healthcare professionals are truly listening in order to tailor care according to the motivation and requests of each patient | 90.6% | N.S.  (p = 0.589) | **YES** |
| {PPR2} Involving the patient’s family and loved ones in care | 28.1% | N.S.  (p = 1.000) | NO |
| {PPR3} Ensuring consistency in the professionals who follow the patient (same doctor, same nurse, etc.) | 90.6% | N.S.  (p = 1.000) | **YES** |
| INFORMATION SYSTEMS | | | |
| {IS1} Easy access for patients to their medical records | 31.3% | N.S.  (p = 0.060) | NO |
| {IS2} Having a dedicated phone support line for registered patients where nurses would have access to the patients’ records | 50.0% | N.S.  (p = 0.479) | NO |
| {IS3} Being able to send and receive information electronically with the clinic (email, texting) regarding health status, test results, notifications for follow-ups, etc. | 56.3% | N.S.  (p = 0.476) | NO |
| {IS4} Having a single, common medical record between all healthcare providers | 81.3% | N.S.  (p = 0.659) | **YES** |

^a^Based on the proportion of panelists rating the item as either 6-“very” or 7-“extremely” important (rating subpanel), or ranking it in the top half of its thematic list (ranking subpanel).

^b^Fisher’s Exact tests, two-tailed significance.

N.S.: not statistically significant (p ≥ 0.05); P: patients; C: clinicians.
