# Supplementary material for: What are the top priorities of patients and clinicians for the organization of primary cardiovascular care in Quebec? A modified e-Delphi study
Source: PLoS One. 2023 Jan 4;18(1):e0280051. doi: 10.1371/journal.pone.0280051 (PMC9812320; doi:10.1371/journal.pone.0280051)
Supplement: Attachment — Submitted filename: Response to reviewers.docx [file pone.0280051.s002.docx]

**Reviewers’ Comments to the Authors:**

**Reviewer 1**

The paper by Del Grande and coauthors covers an important topic and is both interesting and well written. The Delphi process that they used was appropriate to the task at hand.

One can argue whether the findings were surprising. Patients will absolutely want to be seen in a timely fashion by physicians who are personable, communicative, well-organized and up-to-date in their field, all of which I have sought when I have been a patient. The lower priority given by study patients for healthy lifestyle and prevention activities at their primary health care clinics might surprise some clinicians. But patients have other sources for such information; and I could see where, with limited time to see their health care provider, they would wish to focus on the matter at hand rather than hear (yet again) why they need to eat better and exercise more.

Nonetheless, even if at least some of the results might seem expected, the fact that there are divergences of opinion between patients and providers around organizational priorities (among other dimensions) -- ones that are not invariably self-evident -- emphasizes how important such work is to inform clinic and program planning. While the authors list as a limitation the fact that findings relating to the Quebec health care environment might incompletely translate to other settings, I rather suspect that some of the central themes will be broadly noted elsewhere.

Based on this and similar work, it seems that periodically revisiting whether patients and physicians are on the same page should be mandated, especially under circumstances where changes in process or services are being considered by health care providers. Indeed, the authors alluded to the fact that their study was conducted at the time that the COVID infection was especially rampant and disruptive. One of the side effects of the disease has been to put the practice of telemedicine into overdrive. In person clinic visits are returning, but a sizeable number of patient-clinician interactions in my cardiology subspecialty group will continue to be virtual. The work by Del Grande and colleagues shows that we should take no assumptions for granted. In my experience, televideo consultations seem generally well received, especially for routine check-ups of stable patients. But rather than make presumptions, my colleagues and I might do well to survey our patients to know how they feel and have then inform us on how we might do better.

I really do not have any significant criticisms to offer on content or writing style.

**Author response:** We thank Reviewer 1 for the positive review!

Based on his/her relevant comments, we decided to incorporate in our Discussion the idea of revisiting whether patients and clinicians are on the same page when changes in process or services are being considered, by adding (at Lines 469-472^[1]^):

“*Finally, the existence of divergent opinions between patients and clinicians should prompt decision-makers to ensure that both are on the same page whenever changes in care processes or services are being considered.*”

**Reviewer 2**

This is a well-designed and well written study, which I have read with great interest. I have some comments.

**Author response:** We thank Reviewer 2 for the overall positive appraisal of our study. Please find our point-by-point response to Reviewer 2’s comments below.

1) Can you give any reasons why six FMG clinics declined to participate? Line 131-32.

**Author response:** Of course. The reasons were added at Lines 132-137:

“*Of the clinics that did not participate, one declined because it was facing major administrative changes, including a site relocation; one clinic had just come out of a tedious research experience with another team and was reluctant to commit to a new project in the short term; another was in the process of appointing its new research liaison and was unable to coordinate its participation in the project in time; and three did not respond to our invitations and showed no interest when contacted by phone.*”

2a) Were there any changes to the questionnaires as a result of the pre-testing (before round one)?. Line 188-189.

2b) And what professions did the clinicians in the pre-test group have?

**Author response:**

2a) The changes introduced before round one as a result of the pre-test were mainly related to the shortening and simplification of words and sentences.

2b) The clinicians in the pre-test group were both family physicians.

These details were added at Lines 194-195:

“All questionnaires were pretested with nonparticipating patients (n=2) and clinicians (n=2 *family physicians*) to ensure readability and clarity *(shortening and simplification of words and sentences)*, and to estimate completion time.”

3a) Is there any rationale why the limits of 6-7 for rating and correspondingly ranking the item in the top half of its thematic list was set? Line 244-245

3b) In what way are these limits comparable?

**Author response:** We thank Reviewer 2 for allowing us to clarify these important points in the manuscript. These details were added at Lines 252-257, along with two additional references to support our rationale:

“*Focusing on the highest response categories on the rating scale or top half within a ranked list is common in Delphi studies to define consensus.[40,41] The criteria chosen for the rating and ranking subpanels were deemed sufficiently comparable because we expected that most ratings would fall between 4 and 7 on the response scale, given that all the items had been freely elicited as important by panelists beforehand.*”

4) Line 273, was the five nurses registered nurses or assistant nurses? And what profession had the allied health professional?

**Author response:** All five nurses were registered nurses. Unfortunately, we are unable to specify the allied health professional’s profession because he/she did not specify it in the dedicated response field of our study questionnaire.

The nurses’ status was added at Line 284:

“[…] and 16 clinicians (10 family physicians, 5 *registered* nurses and 1 allied healthcare professional).”

5) Among the participating patients almost all had a post-secondary education. I think readers should interpret the result with this in mind, why I suggest adding this as a limitation.

**Author response:** We thank Reviewer 2 for pointing this out and absolutely agree. We have added this limitation at Lines 511-512, when we discuss other limitations of the study:

“*Most of our patient panelists had a postsecondary education, and our results should be interpreted with this in mind.*”

6) Add a footnote below table 1 indicating that patients encompass both patients and faculty patient partners. Line 292.

**Author response:** This was done as suggested by Reviewer 2 (Line 301):

“*Patients encompass both clinic patients and faculty patient partners.*”

7a) Line 294-295, was the number of submitted aspects and the synthesized items equally balanced between patients and clinicians?

7b) Also, it reads: average of 4 per panelist, can this be presented for each group (patient, clinicians) also including min and max?

**Author response:** We thank Reviewer 2 for providing us the opportunity to add these important details in the manuscript.

We added table footnotes in Table 2 to indicate the items that were mentioned only by patient or clinician panelists (at Lines 318-139), and made the following additions at Lines 306-310:

“During round one, 149 potentially important organizational aspects were submitted*:* *78 by patients (mean = 3,9 per patient panelist; min = 1, max = 5) and 71 by clinicians (mean = 4,4 per clinician panelist; min = 3, max = 5).* These were synthesized into 41 mutually exclusive items and grouped under 9 themes (Table 2). *Of those, twenty-seven items had been elicited by both patient and clinician panelists, 9 items by patients only, and 5 by clinicians only.*”

8) Line 361, add percentages along with the p-value 0.014

**Author response:** This was added as suggested by Reviewer 2 (Line 378):

“[…] which was more favored by patients than clinicians (*68.8% vs. 21.4%,* Fisher-exact p = 0.014).”

9) Line 387, the study by Wensing et al. reports patients’ involvement in decisions as one of the most important aspects, which is not so clear from your results. Or which of your identified items and themes reflect that? Please elaborate.

**Author response:** We thank Reviewer 2 for pointing out this potential lack of clarity in our original manuscript. We have added these clarifications at Lines 407-412:

“*Although none of our panelists explicitly used the word ‘decision’ during our first round, the idea of healthcare providers welcoming and heeding patients’ requests to inform care decisions is salient in the number one top shared priority coming out of our study. This item also encapsulates the aspects of humaneness and exploring patients’ needs present in Wensing et al.’s review, underlining the interconnections between these three fundamental aspects of care.*”

1. All line numbers listed in this document refer to the revised manuscript file with Track Changes.
